# Molecular Patterns of MEFV Gene Mutations in Egyptian Patients with Familial Mediterranean Fever: A Retrospective Cohort Study

**DOI:** 10.1155/2019/2578760

**Published:** 2019-02-13

**Authors:** Amal R. Mansour, Ayman El-Shayeb, Nihal El Habachi, Mohamad A. Khodair, Doaa Elwazzan, Nermeen Abdeen, Marwa Said, Riham Ebaid, Noha ElShahawy, Amr Seif, Nadia Zaki

**Affiliations:** ^1^Clinical Pathology Department, Faculty of Medicine, Alexandria University, Egypt; ^2^Mabaret El Asafra Laboratories, Molecular Diagnostics Unit, Egypt; ^3^Tropical Medicine Department, Faculty of Medicine, Alexandria University, Egypt; ^4^Department of Physiology, Faculty of Medicine, Alexandria University, Egypt; ^5^siParadigm Diagnostic Informatics, USA; ^6^Department of Internal Medicine and Hematology, Faculty of Medicine, Alexandria University, Egypt

## Abstract

**Background:**

Familial Mediterranean Fever (FMF) is a hereditary autosomal recessive disease which is mainly seen in the Turks, Armenians, Arabs, and Jews. It is characterized by recurrent episodes of fever, polyserositis, and rash. MEFV gene, encoding pyrin protein, is located on the short arm of chromosome 16. FMF is associated with a broad mutational spectrum in this gene. Certain mutations are more common in particular ethnic groups. To date, different mutations of* MEFV* were observed in studies carried out in different regions worldwide. However, most of these studies did not extensively investigate the Egyptian population, in spite of the high prevalence of FMF in this geographical region.

**Aim:**

To identify the frequency of MEFV gene mutations among the patients who presented with FMF like symptoms and, to characterize the different genetic mutations and their association with increased Amyloid A among Egyptian patients.

**Methods:**

FMF Strip Assay (Vienna Lab Diagnostics, Vienna, Austria) was used. This test is based on reverse hybridization of biotinylated PCR products on immobilized oligonucleotides for mutations and controls in a parallel array of allele-specific oligonucleotides.

**Results:**

Among the 1387 patients presenting with signs and symptoms suggestive of FMF, 793 (57.2%) were of undefined mutations, whereas 594 had MEFV gene mutations. 363 patients (26.2%) were heterozygous mutants, 175 patients (12.6%) were compound heterozygous mutants, and 56 patients (4%) were homozygous mutants. The most commonly encountered gene mutations in heterozygous and homozygous groups were E148Q (38.6%), M694I (18.1%), and V726A (15.8%). The most commonly encountered gene mutations in the compound heterozygous groups were E148Q+M694I observed in 20.6% of the patients, followed by M694I+V726A and M6801+V726A found in 18.9% and 11.4 %, respectively. The most commonly encountered gene mutation associated with abdominal pain, fever, and high serum Amyloid A was E148Q allele* (37.5%)*.

**Conclusions:**

Unlike all previous publications, E148Q allele was found to be the most frequent in the studied patients. Moreover, this allele was associated with increased Amyloid A. 793 patients were free of the 12 studied Mediterranean mutations, which implies the necessity to perform future sequencing studies to reveal other mutations.

## 1. Introduction

FMF is a hereditary autosomal recessive disease with an autoinflammatory nature; it has a worldwide distribution but is mainly seen in the Turks, Armenians, Arabs, and Jews [1]. It is characterized by recurrent episodes of fever, polyserositis, and rash [2]. One of the severe complications of FMF is the development of systemic amyloidosis and renal impairment [3].

Most patients present in childhood or in young adulthood with attacks that often last for 3 days or less and classically recur every few weeks or months [4]. The diagnosis remained predominantly clinical and it was recently confirmed by the MEFV gene mutation analysis [5]. Inflammatory markers such as C-reactive protein, Erythrocyte Sedimentation Rate (ESR), White Blood Count (WBC), and serum Amyloid A evaluation can be helpful for diagnosing and monitoring treatment in patients with FMF [6].

MEFV is the gene responsible for FMF; it is located on the short arm of chromosome 16 [5]. FMF is caused by different mutations in this gene, which encodes a protein named pyrin/marenostrin. Pyrin gene is expressed mostly in neutrophils and in eosinophils, monocytes, dendritic cells, and fibroblasts. The main function of pyrin is its involvement in inflammatory response and in resolving inflammation by the autophagy of regulators of innate immunity, thus controlling inflammation. The variant forms of pyrin that are produced in patients with FMF activity result in inappropriate triggering of neutrophil activation and uncontrolled production of interleukin-1 (IL-1), leading to episodes of inflammation mainly in the peritoneum, pleura, and joints [6]. The variation in the clinical presentation and the development of amyloidosis are influenced by the type of MEFV mutations [2, 6].

There are more than 300 mutation variants located on the MEFV gene. Most of the mutations are in exon 10 between amino acids 680 and 761. In several studies, M694V (valine for methionine at position 694) was found to be the most common mutation. Homozygotes for this mutation may develop more severe clinical manifestations and may be more liable to suffer from amyloidosis. Whereas patients with V726A (alanine for valine at position 726) may be at a lower risk of developing amyloidosis [7]. Certain mutations are more common in particular ethnic groups. However, failure to detect MEFV mutations does not rule out a diagnosis of FMF [8]. The classical treatment of FMF is colchicine. Not only is it effective in preventing attacks of FMF, but it can also protect against the development of amyloidosis. Biologic agents such as Anakinra and Rilonacept can be used as second-line therapy [2, 4, 6].

Although the knowledge of FMF is expanding rapidly and the prevalence of FMF mutations in some Arab populations has already been reported, data on Egyptian population remains very limited.

*Aim of the Work*. Our study aimed to identify the frequency of MEFV gene mutations among the patients who presented with FMF like symptoms. Additionally, we aimed to characterize the different genetic mutations and their association with increased Amyloid A among Egyptian patients.

## 2. Patients and Method

A retrospective cohort study was conducted at Alexandria Main University Hospital and at Mabaret El Asafra Hospital, to study the most frequent gene mutations responsible for FMF symptoms among Egyptians. This study was performed on 1387 patients who presented with symptoms and signs suggestive of FMF between January 2015 and June 2017. The patients were clinically evaluated and screened for the most common 12 MEFV different mutations frequently detected in the Mediterranean region (listed below) using the CE/IVD-labeled FMF Strip Assay (Vienna Lab Diagnostics, Vienna, Austria). This test was based on reverse hybridization of biotinylated PCR products on immobilized oligos for mutations and controls in a parallel array of allele-specific oligonucleotides.

*MEFV Gene Mutations*

- E148Q in exon 2
- P369S in exon 3
- F479L in exon 5
- R761H in exon 10
- I692del
- M680I [G/C]
- M680I [G/A]
- M694V
- M694I
- K695R
- V726A
- A744S

## 3. Results

1387 patients (50.9% males and 49.1% females) were referred to our hospital with symptoms and signs suggestive of FMF. The age of the studied population ranged from 11 to 41 years, with a mean of 22.3 years. Abdominal pain was the most commonly reported complaint, observed in 58.9% of the studied population, followed by fever which was found in 40.7% of the studied population. However, joint pain and elevated serum Amyloid A were reported only in 11.6%  and 8.4 % of the studied group, respectively.

Of all 1387 patients, 594 patients (42.8%) had MEFV gene mutations, while 793 patients (57.2%) were negative for the studied 12 MEFV gene mutations (Table 1).

**Figure 1:**
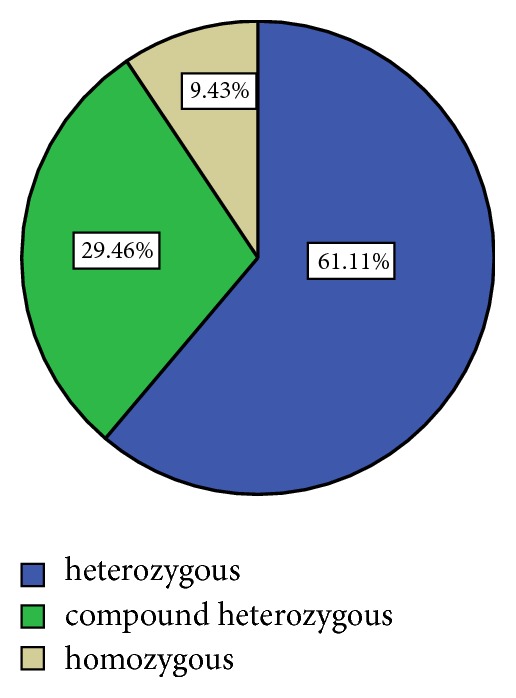
Distribution of MEFV alleles in the studied population.

Among these 594 positive MEFV gene mutations, heterozygous mutations were observed in 363 patients (61.11%), compound heterozygous in 175 patients (29.46%), and homozygous in 56 patients (9.43%) (Figure 1).

**Figure 2:**
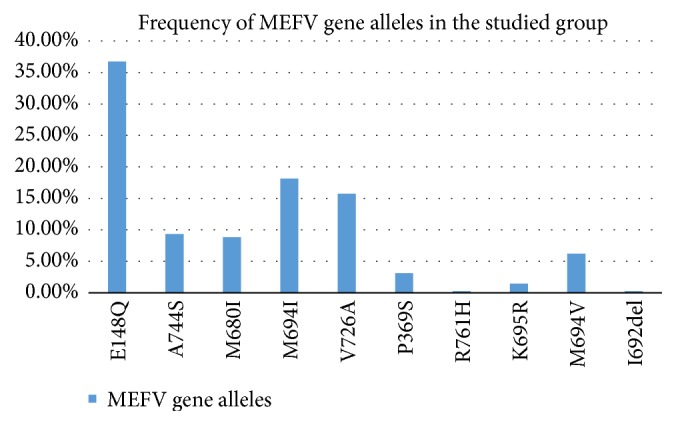
Frequency and spectrum of the common MEFV alleles in the studied population.

*Types of Mutations in the Studied Population*. Heterozygous and homozygous gene mutations were found in 419 patients. The most commonly encountered gene mutations were E148Q (38.6%), M694I (18.1%), and V726A (15.8%) (Figure 2). On the other hand, alleles R761S and I692del showed the least frequencies (0.2%).

The most commonly encountered gene mutations in the compound heterozygous group were E148Q+M694I observed in 20.6% of the compound heterozygous group, followed by M694I+V726A and M6801+V726A found in 18.9% and 11.4 %, respectively.

Among 419 cases of heterozygous and homozygous mutations, 216 were males and 203 were females. The alleles E148Q, M6941, and V726A were of a similar pattern of distribution among males (34.3%, 20.4%, and 18.1%, respectively), and females (39.4%, 15.8%, and 13.3%, respectively).

Elevated serum Amyloid A was found in 8.4% of the patients with positive gene mutations (42 cases heterozygous or homozygous and 8 cases compound heterozygous). In these 42 cases, E148Q had the highest frequency (66.7%) followed by A744S, M6941, and V726A (11.1% for each).

Among the patients who presented with fever, E148Q had also the highest frequency (36.3%) followed by M6941, V726A, and A744S (18.7%, 13.4%, and 11.2%, respectively). Regarding abdominal pain, the most encountered mutant alleles were E148Q, M6941, and V726A (37.5%, 18.5%, and 15.4%).

## 4. Discussion

FMF is an autosomal recessive systemic illness with specific predilection to Mediterranean races. Ethnic differences have been noted in the allele frequency of MEFV gene. In this study, we described the distribution of 12 MEFV gene mutations in a large cohort of Egyptian patients (1387) presenting with signs and symptoms suggestive of FMF.

Our data demonstrated that E148Q mutant allele was the most commonly encountered in the studied patients from Upper and Lower Egypt, with a frequency of 38.6%, followed by M694I, V726A, and A744S mutations with a frequency of 18.1%, 15.8%, and 9.3%, respectively. These results were contradictory to those of other Egyptian research centers that described M694I mutation as being the most frequently detected mutation in all FMF patients. One study was performed in Cairo on just twenty FMF patients [9]. Another research was conducted in Alexandria, on 316 FMF cases, and declared M694I mutation to be the most common as well, with a frequency of 34% followed by E148Q, V726A, M680I, and M694V with frequencies of 22.7%, 15.6%,12.1%, and 7.8%, respectively [10].

Beyond the Egyptian borders within the Mediterranean basin, in a study conducted in Jordan, the most commonly encountered alleles in descending order of frequency were M694V, V726A, and M680I accounting for 38%, 26%, and 10% of cases, respectively [11]. In Morocco, M694V and M694I were the most commonly encountered mutations with frequencies of 49% and 37 %, respectively. These mutations accounted for different proportions of the MEFV mutations in Algeria (5%, 80%), and Tunisia (50%, 25%). M694I mutation was declared as specific to the Arab population from Morocco [12].

In a mixed population of Jewish and Arab children with FMF, the M694V mutation was found in 92% of Jewish patients and in only 30% of the Arab patients [13]. This was in accordance with a study held in Turkey where M694V was the most prevalent mutation reported in 51.4% of cases, followed by M680I mutation (14.4%) and V726A (8.6%) [14]. Similar results were detected in another study conducted in Turkey, where allele frequencies of MEFV mutations were M694V (48%), E148Q (18%), M680I (15%), V726A (12.5%), P369S (3.3%), R761H (0.9), K695R (0.9), E148V (0.9), and A744S (0.5%) [15].

In our study, we found that P369S was the rarest mutation in Egyptian FMF patients. Other published results described R761H mutation to be the least common mutation in Turks and Azeri Turks, and they recommended its incorporation in the mutational scanning analysis for suspected cases of FMF [16]. On the other hand, the F479L mutation, generally reported as rare, was found to be a relatively common mutation in Greek-Cypriot population with an allele frequency of 20.6%. This rare mutation was also reported in Lebanese and Turks, with an allele frequency of 2.9%  and 0.63%, respectively [17, 18].

Discrepancies in the results can be attributed to the differences among ethnic races as well as the differences in the studied sample size. We noted that FMF mutations were significantly higher in males (p=0.003), a finding presumed by other authors to be due to partial penetrance in females [19]. However, a higher frequency of FMF female sex prevalence was observed in other studies performed on Italians and Arabs [20, 21].

Regarding the clinical manifestations, abdominal pain was the most commonly encountered symptom, observed in 58.9% of cases, followed by fever and joint pain, with frequencies of 40.7% and 11.6%, respectively. In another Egyptian study, the clinical manifestations were fever observed in 100% of patients, abdominal pain in 95%, arthritis in 55%, and pleurisy in 40% with no skin rash or pericarditis [9]. In an Arabic group of patients, the most common signs observed were peritonitis (93.7 %), arthritis (33.7 %), and pleurisy (32 %) [19]. In a mixed population of Sephardic Jews, Armenians, Arabs, Turks, French, and others, the main clinical characteristics of the patients were fever (83.3%), abdominal signs (74.0%), thoracic signs (24%), joint signs (50.3%), erysipelas-like erythema (8.2%), splenomegaly (8.5%), and amyloidosis (4%) [21]. In a research that was connducted in Turkey, abdominal pain (76%) and fever (58%) were the two most seen manifestations among patients followed by arthritis (28%) and chest pain (19%) [15]. Another Turkish research detected MEFV gene mutations in forty-three patients with epigastric pain syndrome (57.3%) and the carrier rate was 30.0%. The most common MEFV gene alteration was R202Q (55.8%), followed by E148Q (16.2%), R761H (16.2%), V726A (9.3%), M680I (9.3%), and M694V (4.6%) [22].

Secondary amyloidosis is the most severe complication of FMF. However it is relatively rare. Along the course of Egyptian FMF cases, we noticed that increased Amyloid A was detected with higher frequency among patients with E148Q mutation, and this is contradictory to many previous reports declaring M694V mutation to be associated with clinical severity and amyloidosis as well [23–25]. A study conducted in Mansoura Governorate reported M694V mutation to be the single detected mutation in patients with FMF complicated with amyloidosis, suggesting a hypothetical relation between this mutation and the incidence of amyloidosis in the course of FMF disease. However, this correlation should not be generalized because the authors examined the patients DNA for 4 mutations only, which are M680I, M694I, M694V, and V726A [26].

The overall discrepancy in the prevalent mutation in the MEFV gene can be explained by the racial differences, the geographic distribution, the type of the mutations studied, and the variation in the sample size among the different conducted studies. Finally, we recommend increasing the awareness of the physicians of the high prevalence of FMF in Egypt, the necessity of running molecular tests to identify the exact genotype, and its correlation with disease progression. Moreover, we consider sequencing the MEFV gene in the patients who present with overt symptoms of FMF or have a family history, but proved negative to these 12 mutations.

## Figures and Tables

**Table 1:** Frequency of MEFV gene mutations in the studied group.

Mutation status	Frequency	Percent
Wild	793	57.2
Heterozygous	363	26.2
Compound heterozygous	175	12.6
Homozygous	56	4.0
Total	1387	100.0

## Data Availability

The data used to support the findings of this study are available from the corresponding author upon request.
